# Root Colonization by *Trichoderma atroviride* Triggers Induced Systemic Resistance Primarily Independent of the Chitin-mediated Signaling Pathway in Arabidopsis

**DOI:** 10.1264/jsme2.ME24038

**Published:** 2024-12-27

**Authors:** Ayae Sakai, Hisako Yamagata, Keigo Naito, Mai Yoshioka, Takaya Tominaga, Shinsuke Ifuku, Hironori Kaminaka

**Affiliations:** 1 Department of Agricultural Science, Graduate School of Sustainable Science, Tottori University, Tottori 680–8553, Japan; 2 Faculty of Agriculture, Tottori University, Tottori 680–8553, Japan; 3 The United Graduate School of Agricultural Science, Tottori University, Tottori 680–8553, Japan; 4 Graduate School of Engineering, Tottori University, Tottori 680–8552, Japan; 5 Unused Bioresource Utilization Center, Tottori University, Tottori, 680–8550, Japan

**Keywords:** *Arabidopsis thaliana*, *Alternaria brassicicola*, chitin, induced systemic resistance, *Trichoderma*

## Abstract

Beneficial root endophytic fungi induce systemic responses, growth promotion, and induced systemic resistance (ISR) in colonized host plants. The soil application of chitin, a main component of fungal cell walls, also systemically induces disease resistance. Therefore, chitin recognition and its downstream signaling pathway mediate ISR triggered by beneficial fungi colonizing the root. The present study compared systemic disease resistance and transcriptional responses induced by *Trichoderma*, a representative beneficial root endophytic fungus, and chitin in Arabidopsis. Significant plant growth promotion was observed under root colonization by the three beneficial fungi tested: *Trichoderma atroviride*, *Serendipita indica*, and *S. vermifera*. Only *T. atroviride* and *S. indica* triggered ISR against the necrotrophic fungal pathogen *Alternaria brassicicola*. Induced systemic resistance triggered by *T. atroviride* was compromised in the chitin-receptor mutant, whereas systemic resistance caused by the soil application of chitin was not. A transcriptome ana­lysis demonstrated that chitin-regulated genes were mostly shared with those regulated by *T. atroviride*; however, many of the latter were specific. The commonly enriched gene ontologies for these genes indicated that the *T. atroviride* inoculation and chitin application systemically controlled similar transcriptional responses, mainly associated with cell wall functions. Therefore, *Trichoderma* may trigger ISR primarily independent of the chitin-mediated signaling pathway; however, chitin and *Trichoderma* may systemically induce similar cellular functions aboveground.

Plants have evolved a complex immune system against microbial pathogen infection (Jones and Dangl, 2006). Regarding the surveillance of microbes in host extracellular spaces, conserved microbial elicitors called pathogen-/microbe-associated mole­cular patterns (PAMPs/MAMPs) are recognized by pattern recognition receptors (PRRs) localized on the cell surface (Dodds and Rathjen, 2010). Well-studied PAMP/MAMP-PRR combinations include the flagellin epitope flg22-FLS2 (FLAGELLIN SENSITIVE2), leucine-rich-repeat-type PRR for bacteria and chitin-CERK1 (Chitin Elicitor Receptor Kinase 1), lysin motif (LysM)-type PRR for fungi in the model plant Arabidopsis (Shu *et al.*, 2023). The perception of PAMPs/MAMPs leads to pattern-triggered immunity (PTI), which activates a cellular defense response to restrict microbial invasion (Yuan *et al.*, 2021). Microbial pathogens render the plant susceptible to disease by deploying virulence effectors into host cells to suppress PTI. However, plants recognize pathogen effectors via intracellular nucleotide-binding leucine-rich repeat receptors and induce a strong defense response accompanied by hypersensitive cell death, which is called effecter-triggered immunity (ETI) (Jones and Dangl, 2006).

This plant immune system comprises similar cell-autonomous events to innate immunity in animals; however, plants lack the adaptive immune system in animals (Dodds and Rathjen, 2010). Therefore, plants have developed original systemic immune systems to induce disease resistance against the next pathogen attack in distal parts from the infection site (Pieterse *et al.*, 2014). Systemic acquired resistance (SAR) is a well-studied systemic immunity triggered by PTI and ETI upon pathogen infection. The induction of SAR depends on salicylic acid (SA) and is a long-lasting form of disease resistance against a broad spectrum of (hemi-)biotrophic pathogens (Durrant and Dong, 2004; Vlot *et al.*, 2021). On the other hand, systemic immunity may also be triggered by beneficial or commensal microbes in the plant’s rhizosphere, and is called induced systemic resistance (ISR) (Pieterse *et al.*, 2014; Vlot *et al.*, 2021). Unlike SAR, ISR depends on jasmonic acid and ethylene (ET), which function antagonistically to SA and act mainly against necrotrophic pathogens. Root endophytes include plant growth-promoting rhizobacteria (PGPR), such as *Pseudomonas* spp. and *Bacillus* spp., and plant growth-promoting fungi (PGPF), including *Trichoderma* spp. and *Serendipita* spp., which are known as ISR-inducing rhizospheric microbes (Barazani *et al.*, 2007; Ray *et al.*, 2018; Vlot *et al.*, 2021). As their names suggest, PGPR and PGPF promote plant growth through root colonization. Root colonization by arbuscular mycorrhizal (AM) fungi, which establish mutual symbiosis with approximately 70% of terrestrial plants by exchanging photosynthates for soil-derived mineral nutrients, also triggers ISR (Cameron *et al.*, 2013).

Chitin is a β-1,4-linked linear polymer of *N*-acetylglucosamine and a well-known elicitor derived from fungal cell walls that induces disease resistance (Sharp, 2013). Additionally, the soil application of chitin improves plant growth in various crops, which is considered to be independent of induced disease resistance. We recently reported that supplementing soils with chitin systemically induced disease resistance against necrotrophic fungal pathogens in Arabidopsis, cabbage, strawberry, and rice (Parada *et al.*, 2018; Takagi *et al.*, 2022). Therefore, the application of chitin to soils and beneficial root endophytic fungi induce similar systemic responses in plants, namely, growth promotion and disease resistance. This similarity infers that ISR by beneficial fungi occurs through chitin recognition and its downstream signaling pathway. The present study compared systemic disease resistance induced by a representative PGPF, *Trichoderma*, and chitin against the necrotrophic fungal pathogen, *Alternaria brassicicola*, in *Arabidopsis thaliana*. The evaluation of systemic disease resistance using a chitin-receptor CERK1 mutant indicated that *Trichoderma* triggered ISR primarily independent of the chitin-mediated signaling pathway. The results of a comparative transcriptome ana­lysis showed that a *Trichoderma* inoculation and chitin application in roots systemically controlled similar transcriptional responses aboveground.

## Materials and Methods

### Plant and fungal materials

*A. thaliana* (L) Heynh. accession Col-0 and *cerk1-2* (GABI_096F06) (Miya *et al.*, 2007) were used as the wild type and chitin receptor mutant, respectively. *Trichoderma atroviride* ATCC 20476 and *Serendipita vermifera* MAFF305830 (Warcup, 1988) were maintained on potato dextrose agar (PDA) (Difco) medium at 25°C. *S. indica* WP2 (Sherameti *et al.*, 2005) was maintained on 1/6-strength Czapek Dox agar medium containing 0.8‍ ‍g‍ ‍L^–1^ yeast extract and 15‍ ‍g‍ ‍L^–1^ agar at 25°C.

### Plant growth conditions, fungal inoculation, and chitin application

Arabidopsis seeds were sown on sterilized culture soil (Bestmix No. 3; Nippon Rockwool) and grown under controlled environmental conditions with 14-h light/8-h dark cycles at 23°C for seven weeks by watering 1,000-fold diluted fertilizer (HYPONeX [N–P–K=6–10–5]; Hyponex Japan) weekly.

Regarding the fungal inoculation, pieces of agar medium plugs of maintained fungal strains were placed on YEPG medium (Yeast extract 3‍ ‍g‍ ‍L^–1^, Polypeptone 3‍ ‍g‍ ‍L^–1^, and D-glucose 20‍ ‍g‍ ‍L^–1^) and cultured using a rotary shaker at 25°C in the dark for two weeks. After removing the liquid medium, the harvested mycelia were homogenized with a blender (Nissei) at 10,000‍ ‍rpm for 10 s. Distilled water was added to prepare mycelial suspensions at the indicated concentrations. Five milliliters of each mycelial suspension was irrigated into soils two weeks after sowing, and Arabidopsis seedlings were grown for an additional five weeks. The water dispersion of chitin nanofibers (CNF) (MARINE NANO-FIBER CN-01), which are directly produced from chitin powder by physically grinding microfibrils (nanofibrillation) and may be used like a water solution (Ifuku and Saimoto, 2012), was purchased from Marine Nano-fiber and used for the chitin treatment unless otherwise indicated. Chitin oligosaccharide (CO) mixture powder (NA-COS-Y: Yaizu Suisankagaku Industry), containing CO with a degree of polymerization of 2–6, was also used. Upon preparation, the culture soil was mixed with an equal volume of 0.1% (w/v) CNF water dispersion or 0.1% (w/v) CO water solution before sowing, based on our previous study (Kaminaka *et al.*, 2020). Distilled water was used for control experiments.

### Fluorescent staining of fungal hyphae in Arabidopsis roots

Harvested Arabidopsis roots were fixed in 70% ethanol overnight. After removing ethanol, 5% KOH was added, and samples were heated at 90°C for 30‍ ‍min. Root samples were transferred into 1% HCl for neutralization for 5‍ ‍min, washed with PBS buffer, and incubated with 5‍ ‍μg mL^–1^ of WGA-Alexa Fluor 488 (Thermo Fisher Scientific) in the dark for 10‍ ‍min. Roots were washed with PBS again, and 20% TOMEI (Tokyo Chemical Industry) was added to clear tissues. Stained roots were observed under a fluorescent microscope (DM2500; Leica) with the excitation filter L5, and photo images were obtained with the equipped digital camera (DFC310; Leica).

### Detection of root-colonized beneficial fungi by PCR

Harvested Arabidopsis roots were subjected to the extraction of genomic DNA (gDNA) using RBC Genomic DNA Extraction Kit Mini (Plant) (RBC Bioscience). The concentration of extracted gDNA was measured using the DS-11 spectrophotometer (DeNovix). Each PCR reaction mixture was prepared in a final volume of 25‍ ‍μL containing 5.5‍ ‍ng gDNA, 12.5‍ ‍μL of KOD One PCR Master Mix (TOYOBO), and 1.5‍ ‍μL of 5‍ ‍μM of each primer. PCR was conducted on the T100 Thermal Cycler (BIO-RAD). Detailed information on the PCR reaction, including the target genes, the sequences of the primers used, the amplicon size, and PCR reaction conditions, was provided in Table S1. PCR products were separated by 1.5% agarose gel electrophoresis with the FastGene 100 bp DNA Maker (NIPPON GENE), and images of ethidium bromide-stained gels were taken using the GelDoc GO Imaging system (BIO-RAD).

### Disease resistance assay

Spores of the fungal pathogen *A. brassicicola* strain O-264, a causal agent of black leaf spot on Brassica plants, were prepared and inoculated on Arabidopsis leaves as previously reported (Parada *et al.*, 2018). Ten microliters of a conidial suspension (5.0×10^5^ spores mL^–1^) was inoculated onto each leaf. The diameter of the lesions that emerged on leaves was measured four d after the inoculation using ImageJ ver.1.53a.

### RNA-sequencing and data ana­lysis

Approximately 100‍ ‍mg of randomly selected leaves from at least three individual seven-week-old Arabidopsis seedlings inoculated with *T. atroviride* or treated with chitin were used to prepare the sequencing library. Total RNA preparation was conducted according to Tominaga *et al.* (2021). The preparation of sequencing libraries and sequencing with strand-specific and paired-end reads (150 bp) by DNBSEQ-T7RS was performed by Genome-Lead.

Low-quality reads (<QV30) and adapter sequences of the raw reads obtained were removed by fastp (Chen *et al.*, 2018) and mapped onto the sequence of the Arabidopsis reference genome TAIR10.43 (https://www.arabidopsis.org/) by the RNA-sequencing aligner STAR ver.2.6.1d (Dobin *et al.*, 2013) (Table S2). Data were processed with featureCounts ver.2.0.1 (Liao *et al.*, 2014) to obtain gene expression count data. Count data in different library sizes were normalized by the trimmed mean of the *M*-values normalization method, and differentially expressed genes (DEGs) were identified by comparing control and *T. atroviride*-inoculated or chitin-treated plants using edgeR ver.4.2.1 (Robinson *et al.*, 2010). The list of DEGs was extracted by a false discovery rate (FDR) cut-off <0.05. Venn diagrams were generated using the Venn diagram website (https://bioinformatics.psb.ugent.be/webtools/Venn). The gene ontology (GO) enrichment ana­lysis was conducted using Shiny GO 0.77 (Ge *et al.*, 2020), and dot plots were drawn using “ggplot2” and “ggpubr” packages in R (ver.4.3.1).

### RNA-sequencing data accession number

The raw read data obtained by RNA-sequencing were deposited in the DNA Data Bank of Japan under the BioProject accession number PRJDB17932.

### Statistical ana­lysis

Lesion diameters caused by the *A. brassicicola* inoculation were compared to control plants, and the significance of differences in the results obtained was analyzed using the Student’s *t*-test and Microsoft Excel (ver. 2312). All pathogen inoculation tests were conducted at least three times with more than three different plants for each treatment and genotype.

## Results

### Growth promotion and ISR by the beneficial fungal colonization of Arabidopsis

**Fig. 1.**
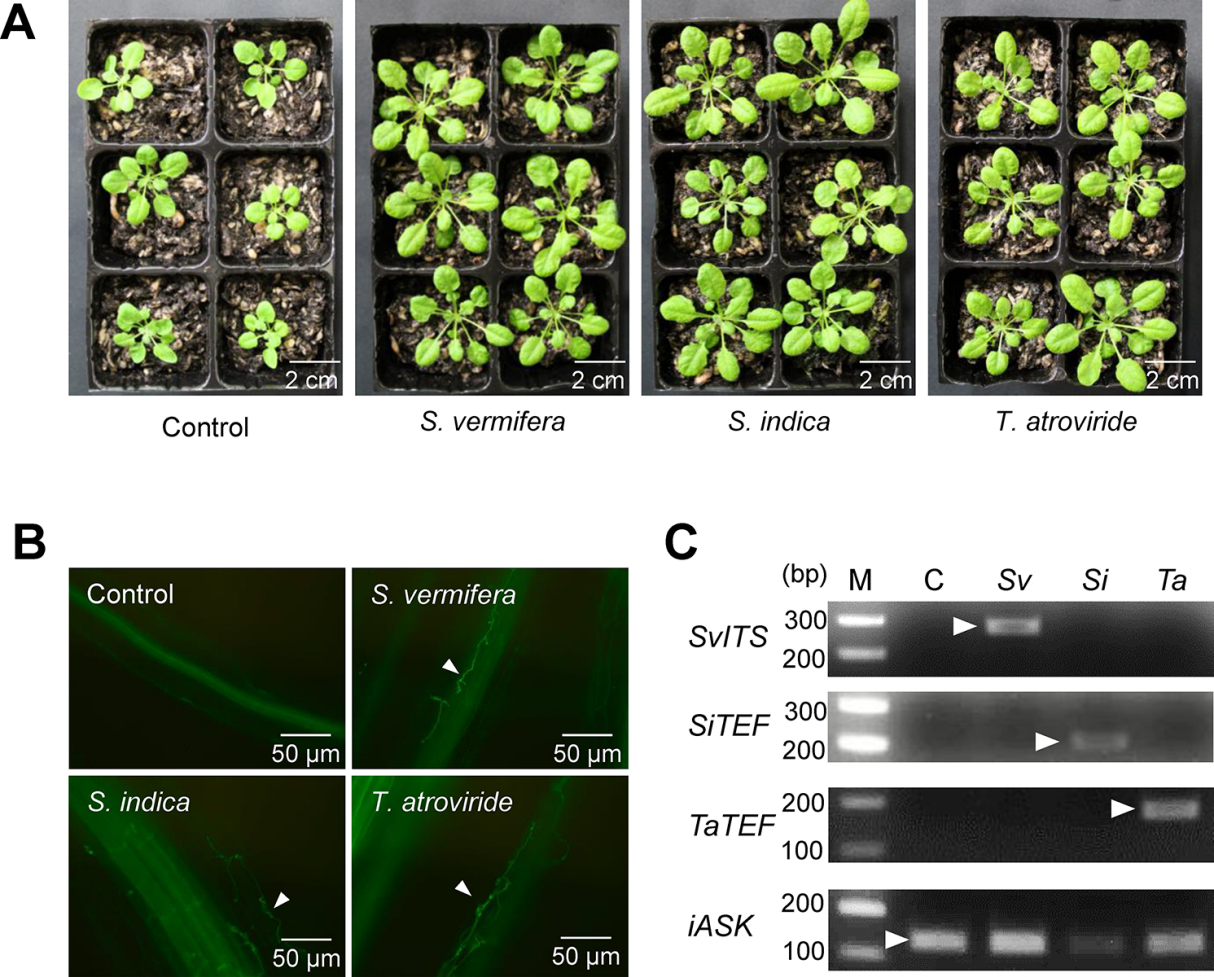
Growth promotion of Arabidopsis seedlings colonized by beneficial fungi. (A) Photos of Arabidopsis seedlings grown for seven weeks on soil irrigated with hyphal homogenates (50‍ ‍mg fresh weight [FW] mL^–1^) of *Serendipita vermifera*, *S. indica*, and *Trichoderma atroviride*. (B) Fluorescent images of inoculated fungal hyphae stained with WGA-Alexa Fluor 488 in Arabidopsis roots. The white arrowheads indicate fungal hyphae. (C) Detection of colonized beneficial fungi in Arabidopsis roots by PCR using gene-specific primers of colonized fungi and Arabidopsis: *S. vermifera 5.8S coding sequence and highly variable ITS2 region of ribosomal DNA* (*SvITS*), *S. indica translation elongation factor 1 alpha* (*SiTEF*), *T. atroviride translation elongation factor 1 alpha* (*TaTEF*), and *Arabidopsis thaliana SHAGGY-related kinase 11* (*iASK*). Arrowheads indicate the bands corresponding to the amplicon size. M: Molecular marker, C: Control, *Sv*: *S. vermifera*, *Si*: *S. indica*, *Ta*: *T. atroviride*.

Root endophytes promote plant growth and systemically induce disease resistance, and are called PGPR and PGPF (Pieterse *et al.*, 2014; Vlot *et al.*, 2021). In Arabidopsis, *Trichoderma* and *Serendipita* species are known as PGPF (Lahrmann and Zuccaro, 2012; González-Pérez *et al.*, 2018). To compare the effects of beneficial fungi inoculated on Arabidopsis seedlings, *T. atroviride*, *S. indica*, and *S. vermifera* were inoculated by irrigating the soil with mycelial suspensions. The growth of Arabidopsis seedlings inoculated with these three fungi was significantly better than that of non-inoculated seedlings at five weeks post-inoculation (Fig. 1A). Fungal colonization was confirmed by hyphal staining and PCR only in the roots of fungus-inoculated seedlings (Fig. 1B and C). These results indicate that root colonization by these beneficial fungi promoted plant growth.

**Fig. 2.**
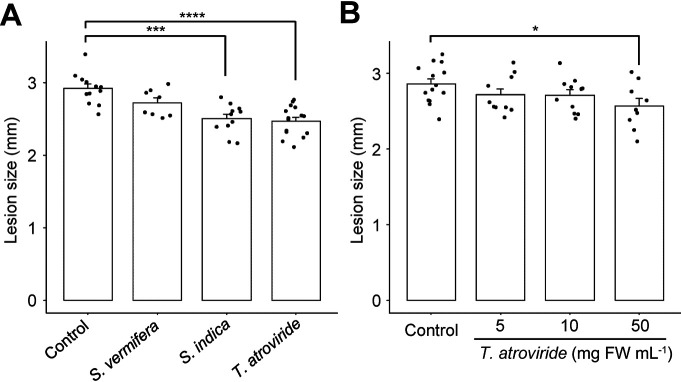
Induced systemic resistance against a necrotrophic pathogen after an inoculation with beneficial fungi. (A) Lesion size on Arabidopsis seedling leaves (grown as in Fig. 1) inoculated with 10‍ ‍μL of the *Alternaria brassicicola* suspension (5.0×10^5^ spores mL^–1^). The lesion was measured four d after the inoculation. (B) The effects of the inoculum concentration (mg fresh weight [FW] mL^–1^) for the *Trichoderma atroviride* inoculation, conducted as in (A). Bars and error bars indicate means and standard errors, and asterisks indicate significant differences (the Student’s *t*-test: *P<0.05, ***P<0.001, ****P<0.0001; *n*≥8).

Since beneficial fungi cause ISR mainly against necrotrophic pathogens (Pieterse *et al.*, 2014; Vlot *et al.*, 2021), we exami­ned disease resistance against the necrotrophic fungal pathogen *A. brassicicola*, a causal agent of black leaf spot on Brassica plants, in the leaves of beneficial fungus-inoculated seedlings. Root colonization by *T. atroviride* and *S. indica* significantly reduced lesion sizes. In contrast, no significant differences in lesion formation were observed between *S. vermifera*-colonized seedlings and control seedlings (Fig. 2A). Since *T. atroviride* displayed significantly more ISR than *S. indica*, it was selected for the subsequent experiment to optimize the fungal inoculum concentration. Only the concentration used in the previous experiment (50‍ ‍mg FW mL^–1^), not lower concentrations, led to significantly smaller lesions caused by the *A. brassicicola* infection (Fig. 2B). Therefore, the concentration of the *T. atroviride* inoculum of 50‍ ‍mg FW mL^–1^ was used in further experiments.

### Chitin receptor CERK1 functions in disease resistance systemically induced by *T. atroviride* and chitin

**Fig. 3.**
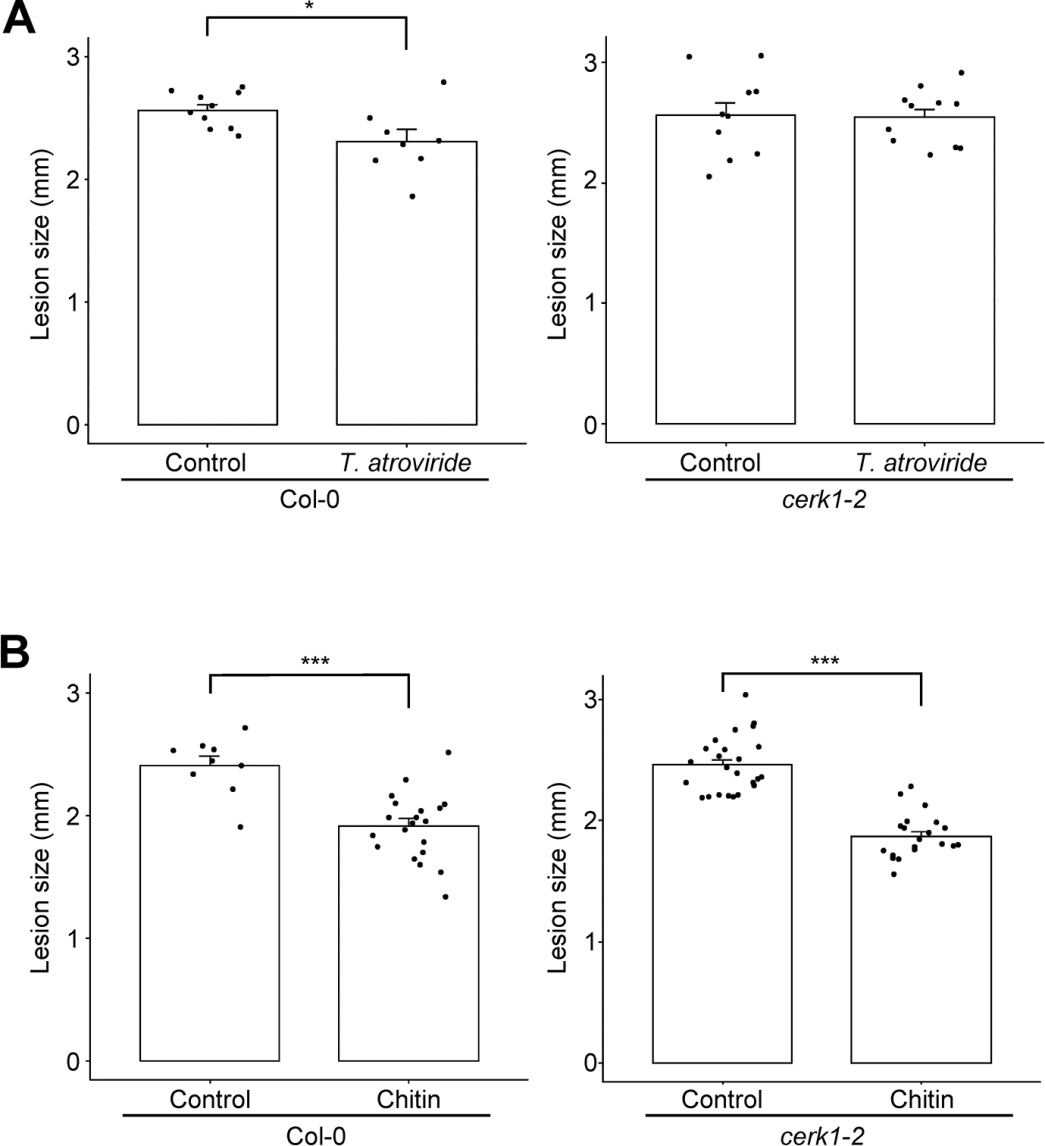
Effects of chitin receptor deficiency on induced systemic resistance against a necrotrophic pathogen. Disease resistance against *Alternaria brassicicola* on the leaves of wild-type (Col-0) and *cerk1-2* seedlings (A) inoculated with *Trichoderma atroviride* and (B) treated with chitin, as conducted in Fig. 2. Bars and error bars indicate means and standard errors, and asterisks indicate significant differences (the Student’s *t*-test: *P<0.05, ***P<0.001; *n*≥8).

Chitin is a PAMP/MAMP used by plants to sense the presence of fungi in intracellular spaces (Shu *et al.*, 2023). Similar to beneficial fungal colonization, the application of chitin to soils has been shown to promote plant growth and induce systemic resistance (Parada *et al.*, 2018; Kaminaka *et al.*, 2020; Takagi *et al.*, 2022). Therefore, the involvement of chitin in ISR triggered by *T. atroviride* root colonization was investigated using the chitin receptor LysM-type PRR CERK1-deficient mutant *cerk1-2* (Miya *et al.*, 2007). ISR against *A. brassicicola* observed in wild-type plants inoculated with *T. atroviride* was compromised in *cerk1-2* (Fig. 3A). In the present study, chitin nanofibers were used as chitin because they induce a more robust plant response than other chitins through the rapid and massive production of CO by chitinase (Egusa *et al.*, 2015; Kaminaka *et al.*, 2020). Systemic disease resistance against *A. brassicicola* was significantly induced, even in *cerk1-2*, by the application of chitin to soils (Fig. 3B). The application of CO to soils also showed a similar result (Fig. S1), which indicated that resistance systemically induced by any form of chitin is independent of CERK1 functions. These results reveal that ISR triggered by *T. atroviride* root colonization may be regulated differently from, and potentially independent of, chitin-triggered systemic disease resistance in Arabidopsis.

### Comparative transcriptome ana­lysis of *T. atroviride*-inoculated or chitin-treated Arabidopsis seedlings

**Fig. 4.**
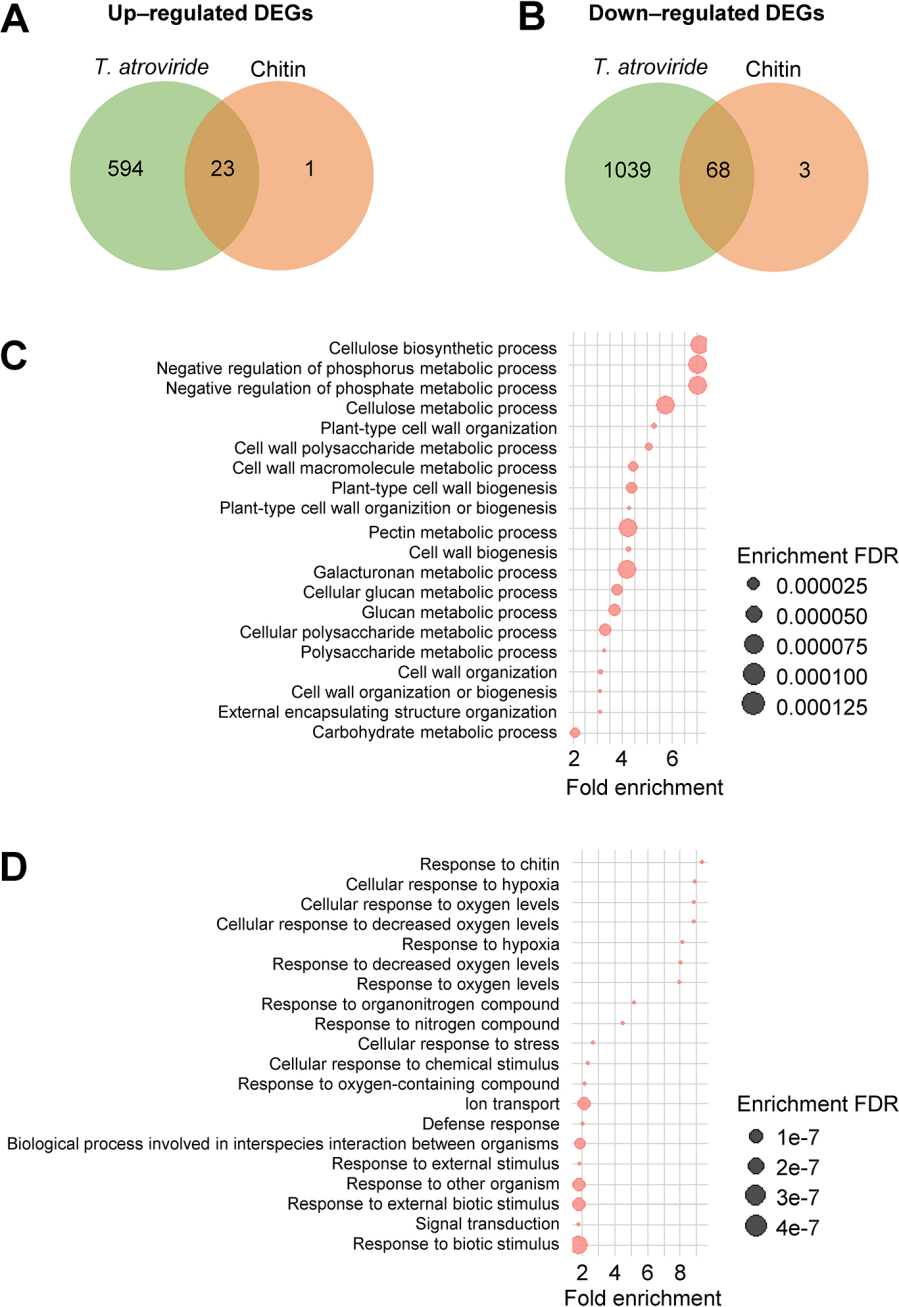
Transcriptome ana­lysis of leaves of Arabidopsis seedlings inoculated with *Trichoderma atroviride* and treated with chitin. Venn diagrams of up-regulated (A) and down-regulated (B) differentially expressed genes (DEGs) identified by a false discovery rate (FDR) cut-off <0.05. The top 20 enriched biological process gene ontology (GO) terms with the lowest FDR values for up-regulated (C) and down-regulated (D) DEGs upon the *T. atroviride* inoculation. The circle size indicates the FDR value. The complete list of enriched GO terms is presented in Table S5.

To elucidate the mole­cular mechanisms underlying ISR caused by the root colonization of *T. atroviride*, Arabidopsis roots were inoculated with *T. atroviride* or treated with chitin, and seedling leaves were used for RNA-sequencing to elucidate the mole­cular mechanisms responsible for ISR caused by *T. atroviride*. Compared with control seedlings, 1,724 DEGs were identified in *T. atroviride*-inoculated seedlings, including 617 up-regulated and 1,107 down-regulated DEGs (Fig. 4A, B and Table S3). We identified 95 DEGs in chitin-treated seedlings, including 24 up-regulated and 71 down-regulated DEGs (Fig. 4A, B and Table S4). More than 95% of DEGs in chitin-treated seedlings were shared with those in *T. atroviride*-inoculated seedlings (Fig. 4A and B).

A GO enrichment ana­lysis of DEGs identified in the leaves of *T. atroviride*-inoculated seedlings was then performed. Regarding up-regulated DEGs, GO terms associated with cell wall functions (*e.g.*, “Cellulose biosynthesis/metabolic process”, “Plant-type cell wall organization or biogenesis”, and “Cell wall polysaccharide/macromolecule metabolic process”) were dominantly overrepresented in the biological process category (Fig. 4C and Table S5) and were also identified as enriched GO terms for up-regulated DEGs in chitin-treated seedling leaves (Fig. S2A and Table S6). The enrichment for categories related to the negative regulation of the phosphorus metabolic process, kinase inhibitor activity, and endocytic pathway (*e.g.*, endosome, Golgi, or vesicle) was also indicated (Fig. 4C and Table S5). Regarding down-regulated DEGs, the GO term “Response to chitin” was highly enriched, and overrepresented GO terms associated with cellular responses to oxygen levels, transcription factors, and NAD/NAD(P)+ nucleoside activity were also found (Fig. 4D and Table S5). These GO terms were also overrepresented for down-regulated DEGs in chitin-treated seedlings (Fig. S2B and Table S6).

## Discussion

*Trichoderma* is widely used as a biocontrol agent mainly against soil-borne diseases in various crops through its mycoparasitism and secretomes, including volatile organic compounds (VOCs), cell wall-degrading enzymes (CDWEs), reactive oxygen species, and antimicrobial secondary metabolites (Alfiky and Weisskopf, 2021; Yao *et al.*, 2023). Additionally, *Trichoderma* induces systemic responses in host plants, including growth promotion and disease resistance known as ISR. The combination of these functions may cause the biocontrol effects of *Trichoderma*; however, available knowledge on each function needs to be improved. To obtain novel insights into plant–*Trichoderma* interactions, we investigated the mole­cular mechanisms underlying *Trichoderma*-ISR by focusing on the involvement of chitin—recognition and signaling—in Arabidopsis. The ana­lysis of systemic disease resistance in chitin receptor-deficient mutants revealed that *Trichoderma* triggered systemic resistance against necrotrophic pathogens primarily independent of the chitin-mediated signaling pathway.

The root colonization of all exami­ned endophytic fungi, *T. atroviride*, *S. indica*, and *S. vermifera*, promoted plant growth, which is consistent with previous findings from various plants (Lahrmann and Zuccaro, 2012; Ray *et al.*, 2018; Alfiky and Weisskopf, 2021). In contrast, only *T. atroviride* and *S. indica* significantly increased disease resistance against the necrotrophic pathogen, *A. brassicicola*, in the leaves of root-colonized Arabidopsis seedlings. ISR by the root colonization of *T. atroviride* and *S. indicia* in Arabidopsis has been reported (Salas-Marina *et al.*, 2011; Lahrmann and Zuccaro, 2012), but not for *S. vermifera*. Sarkar *et al.* (2019) revealed the mycoparasitism of *S. vermifera* against *Bipolaris sorokiniana*, a causal agent of spot blotch and common root rot diseases, by mainly reducing the pathogen’s root infection in barley. They also suggested disease resistance systemically induced by *S. vermifera* in roots. Therefore, *S. vermifera* may be able to cause weak ISR, which may not be sufficient for detection using our Arabidopsis pathosystem.

ISR in Arabidopsis has been extensively exami­ned using PGPR, such as *Pseudomonas* and *Bacillus* species (Pieterse *et al.*, 2014; Vlot *et al.*, 2021). However, knowledge of ISR by beneficial fungi is limited due to AM fungi being a non-host. The present study demonstrated that the function of the chitin-receptor CERK1 was required for ISR by *Trichoderma* in Arabidopsis. The ectomycorrhizal fungus *Laccaria bicolor* triggered ISR against the cabbage looper *Trichoplusia ni* and induced systemic susceptibility against the hemi-biotrophic bacterial pathogen *Pseudomonas syringae* pv. *tomato* DC3000 in non-host Arabidopsis plants in a CERK1-dependent manner (Vishwanathan *et al.*, 2020). Since the treatments of heat-killed *L. bicolor* and chitin also systemically induced disease resistance, the authors proposed that ISR without a symbiotic association may be triggered by the root perception of PAMPs/MAMPs, which was supported by our previous findings (Takagi *et al.*, 2022). In rice, supplementing soils with chitin systemically induced disease resistance against the necrotrophic pathogen *Bipolaris oryzae* through the function of LysM-type PRRs, OsCERK1, and OsCEBiP. In contrast, chitin-ISR in Arabidopsis was not mediated by CERK1 in this study, which indicated that the general model for chitin perception mediated by CERK1 function does not apply to chitin-induced systemic disease resistance in Arabidopsis. Although the mechanism for chitin perception by LysM-type PRRs in Arabidopsis is similar to that in rice, CERK1 functions differ in terms of its binding ability to chitin; unlike rice CERK1 (OsCERK1), Arabidopsis CERK1 binds to CO (Liu *et al.*, 2012; Shinya *et al.*, 2012; Yang *et al.*, 2022). Therefore, the difference in the chitin perception mechanism may be explained by the different requirements of CERK1 functions for chitin-ISR between Arabidopsis and rice. To address this point, Arabidopsis LysM-type PRR(s) involved in chitin-ISR and *Trichoderma*-induced ISR need to be characterized using loss-of-function mutants, which will be conducted in a subsequent study.

The transcriptome ana­lysis revealed that most DEGs identified in chitin-treated seedlings were shared with those in* Trichoderma*-inoculated seedlings, indicating the minor or no contribution of chitin-triggered functions in *Trichoderma*-induced systemic responses. Additionally, 94% of DEGs identified in *Trichoderma*-inoculated seedlings were specific; therefore, *Trichoderma*-specific transcriptional responses may contribute to systemic responses, including ISR and growth promotion. The GO terms involved in cell wall functions were mainly overrepresented by the GO enrichment ana­lysis of up-regulated DEGs in *Trichoderma*-inoculated seedlings. Recent studies revealed the involvement and importance of cell wall functions (*e.g.*, cell wall biogenesis, composition, and integrity) in the induction of disease resistance (Bacete *et al.*, 2018; Molina *et al.*, 2021; Baez *et al.*, 2022). Therefore, the modulation of cell wall conditions by transcriptional changes may be a major cellular event inducing disease resistance aboveground in *Trichoderma*-induced systemic responses. In our previous study, the GO terms associated with cell wall functions were also enriched in the leaves of rice seedlings grown on chitin-supplemented soils (Takagi *et al.*, 2022). However, unlike in Arabidopsis, the genes involved in cell wall functions were down-regulated in rice. These opposite aboveground transcriptional responses may also describe the different requirements of CERK1 functions for chitin-ISR between Arabidopsis and rice.

**Fig. 5.**
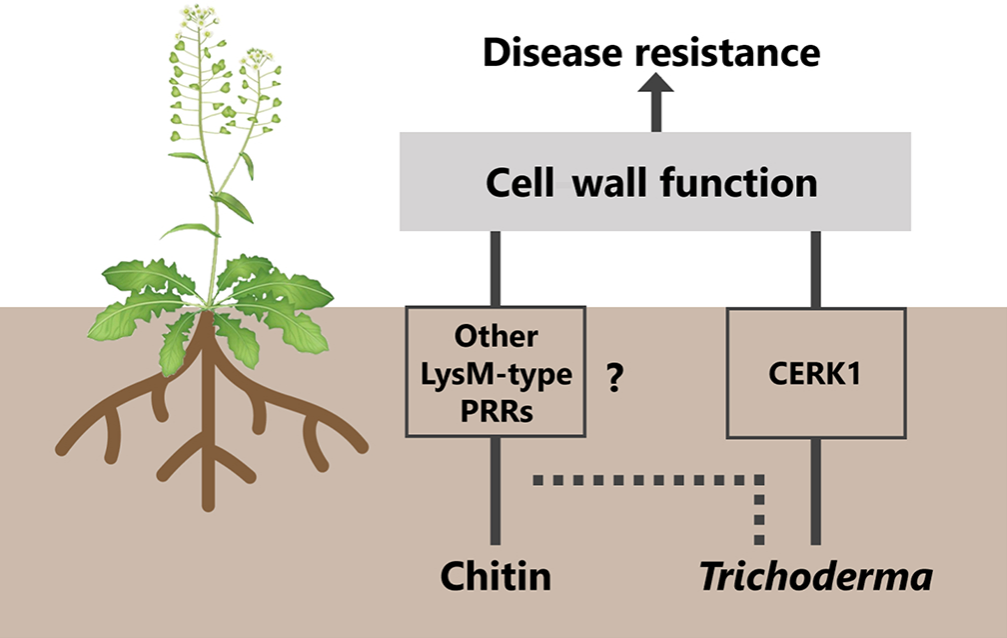
Proposed model for chitin- and *Trichoderma*-induced systemic resistance (ISR) in Arabidopsis. Chitin supplementation into soils and root colonization by *Trichoderma atroviride* systemically up-regulate cell wall-related genes in leaves and induce disease resistance against the necrotrophic fungal pathogen *Alternaria brassicicola*. The function of Chitin Elicitor Receptor Kinase 1 (CERK1), the lysin motif (LysM)-type pattern recognition receptor required for chitin perception (Miya *et al.*, 2007), is required for ISR by *Trichoderma*, whereas CERK1 is not involved in ISR by chitin. Therefore, chitin and *Trichoderma* may systemically modulate similar cellular functions for aboveground ISR. However, *Trichoderma* induces systemic responses primarily independent of the chitin-mediated signaling pathway.

The enriched GO terms for DEGs were similar between the *Trichoderma* inoculation and chitin treatment. Therefore, this inoculation/treatment may induce systemic responses by modulating similar cellular functions mainly associated with aboveground cell wall functions, even if the requirement of LysM-type PRRs is different (Fig. 5). In the present study, we identified a specific signaling pathway mediated by CERK1 in ISR triggered by *Trichoderma*, which is primarily independent of the chitin-mediated signaling pathway. Plant hormones and secretomes, including effectors, VOCs, and CDWEs, also participate in ISR by *Trichoderma* (Alfiky and Weisskopf, 2021). Therefore, we plan to conduct further studies with a focus on the involvement of these molecules in order to elucidate the mechanisms underlying the *Trichoderma*-specific signaling pathway systemically inducing disease resistance in Arabidopsis.

## Citation

Sakai, A., Yamagata, H., Naito, K., Yoshioka, M., Tominaga, T., Ifuku, S., and Kaminaka, H. (2024) Root Colonization by *Trichoderma atroviride* Triggers Induced Systemic Resistance Primarily Independent of the Chitin-mediated Signaling Pathway in Arabidopsis. *Microbes Environ ***39**: ME24038.

https://doi.org/10.1264/jsme2.ME24038

## Supplementary Material

Supplementary Material 1

Supplementary Material 2

## References

[B1] Alfiky, A., and Weisskopf, L. (2021) Deciphering *Trichoderma*–plant–pathogen interactions for better development of biocontrol applications. J Fungi 7: 1–18. 10.3390/jof7010061 PMC7830842 33477406

[B2] Bacete, L., Mélida, H., Miedes, E., and Molina, A. (2018) Plant cell wall-mediated immunity: cell wall changes trigger disease resistance responses. Plant J 93: 614–636. 29266460 10.1111/tpj.13807

[B3] Baez, L.A., Tichá, T., and Hamann, T. (2022) Cell wall integrity regulation across plant species. Plant Mol Biol 109: 483–504. 35674976 10.1007/s11103-022-01284-7 PMC9213367

[B4] Barazani, O., von Dahl, C.C., and Baldwin, I.T. (2007) *Sebacina vermifera* promotes the growth and fitness of *Nicotiana attenuata* by inhibiting ethylene signaling. Plant Physiol 144: 1223–1232. 17416638 10.1104/pp.107.097543 PMC1914189

[B5] Cameron, D.D., Neal, A.L., van Wees, S.C.M., and Ton, J. (2013) Mycorrhiza-induced resistance: more than the sum of its parts? Trends Plant Sci 18: 539–545. 23871659 10.1016/j.tplants.2013.06.004 PMC4194313

[B6] Chen, S., Zhou, Y., Chen, Y., and Gu, J. (2018) fastp: an ultra-fast all-in-one FASTQ preprocessor. Bioinformatics 34: i884–i890. 30423086 10.1093/bioinformatics/bty560 PMC6129281

[B8] Dobin, A., Davis, C.A., Schlesinger, F., Drenkow, J., Zaleski, C., Jha, S., et al. (2013) STAR: Ultrafast universal RNA-seq aligner. Bioinformatics 29: 15–21. 23104886 10.1093/bioinformatics/bts635 PMC3530905

[B9] Dodds, P.N., and Rathjen, J.P. (2010) Plant immunity: towards an integrated view of plant–pathogen interactions. Nat Rev Genet 11: 539–548. 20585331 10.1038/nrg2812

[B10] Durrant, W.E., and Dong, X. (2004) Systemic acquired resistance. Annu Rev Phytopathol 42: 185–209. 15283665 10.1146/annurev.phyto.42.040803.140421

[B11] Egusa, M., Matsui, H., Urakami, T., Okuda, S., Ifuku, S., Nakagami, H., and Kaminaka, H. (2015) Chitin nanofiber elucidates the elicitor activity of polymeric chitin in plants. Front Plant Sci 6: 1098. 26697049 10.3389/fpls.2015.01098 PMC4673310

[B13] Ge, S.X., Jung, D., and Yao, R. (2020) ShinyGO: a graphical gene-set enrichment tool for animals and plants. Bioinformatics 36: 2628–2629. 31882993 10.1093/bioinformatics/btz931 PMC7178415

[B14] González-Pérez, E., Ortega-Amaro, M.A., Salazar-Badillo, F.B., Bautista, E., Douterlungne, D., and Jiménez-Bremont, J.F. (2018) The Arabidopsis-Trichoderma interaction reveals that the fungal growth medium is an important factor in plant growth induction. Sci Rep 8: 16427. 30401880 10.1038/s41598-018-34500-w PMC6219587

[B16] Ifuku, S., and Saimoto, H. (2012) Chitin nanofibers: preparations, modifications, and applications. Nanoscale 4: 3308–3318. 22539071 10.1039/c2nr30383c

[B17] Jones, J.D.G., and Dangl, J.L. (2006) The plant immune system. Nature 444: 323–329. 17108957 10.1038/nature05286

[B18] Kaminaka, H., Miura, C., Isowa, Y., Tominaga, T., Gonnami, M., Egusa, M., et al. (2020) Nanofibrillation is an effective method to produce chitin derivatives for induction of plant responses in soybean. Plants 9: 810. 32605205 10.3390/plants9070810 PMC7411678

[B20] Lahrmann, U., and Zuccaro, A. (2012) *Opprimo ergo sum*—evasion and suppression in the root endophytic fungus *Piriformospora indica*. Mol Plant Microbe Interact 25: 727–737. 22352718 10.1094/MPMI-11-11-0291

[B21] Liao, Y., Smyth, G.K., and Shi, W. (2014) featureCounts: an efficient general purpose program for assigning sequence reads to genomic features. Bioinformatics 30: 923–930. 24227677 10.1093/bioinformatics/btt656

[B22] Liu, T., Liu, Z., Song, C., Hu, Y., Han, Z., She, J., et al. (2012) Chitin-induced dimerization activates a plant immune receptor. Science 336: 1160–1164. 22654057 10.1126/science.1218867

[B23] Miya, A., Albert, P., Shinya, T., Desaki, Y., Ichimura, K., Shirasu, K., et al. (2007) CERK1, a LysM receptor kinase, is essential for chitin elicitor signaling in *Arabidopsis*. Proc Natl Acad Sci U S A 104: 19613–19618. 18042724 10.1073/pnas.0705147104 PMC2148337

[B24] Molina, A., Miedes, E., Bacete, L., Rodríguez, T., Mélida, H., Denancé, N., et al. (2021) *Arabidopsis* cell wall composition determines disease resistance specificity and fitness. Proc Natl Acad Sci U S A 118: e2010243118. 33509925 10.1073/pnas.2010243118 PMC7865177

[B25] Parada, R.Y., Egusa, M., Aklog, Y.F., Miura, C., Ifuku, S., and Kaminaka, H. (2018) Optimization of nanofibrillation degree of chitin for induction of plant disease resistance: elicitor activity and systemic resistance induced by chitin nanofiber in cabbage and strawberry. Int J Biol Macromol 118: 2185–2192. 30021137 10.1016/j.ijbiomac.2018.07.089

[B26] Pieterse, C.M.J., Zamioudis, C., Berendsen, R.L., Weller, D.M., Van Wees, S.C.M., and Bakker, P.A.H.M. (2014) Induced systemic resistance by beneficial microbes. Annu Rev Phytopathol 52: 347–375. 24906124 10.1146/annurev-phyto-082712-102340

[B28] Ray, P., Chi, M.-H., Guo, Y., Chen, C., Adam, C., Kuo, A., et al. (2018) Genome sequence of the plant growth promoting fungus *Serendipita vermifera* subsp. *bescii*: the first native strain from North America. Phytobiomes J 2: 62–63.

[B29] Robinson, M.D., McCarthy, D.J., and Smyth, G.K. (2010) edgeR: a bioconductor package for differential expression ana­lysis of digital gene expression data. Bioinformatics 26: 139–140. 19910308 10.1093/bioinformatics/btp616 PMC2796818

[B30] Salas-Marina, M.A., Silva-Flores, M.A., Uresti-Rivera, E.E., Castro-Longoria, E., Herrera-Estrella, A., and Casas-Flores, S. (2011) Colonization of *Arabidopsis* roots by *Trichoderma atroviride* promotes growth and enhances systemic disease resistance through jasmonic acid/ethylene and salicylic acid pathways. Eur J Plant Pathol 131: 15–26.

[B31] Sarkar, D., Rovenich, H., Jeena, G., Nizam, S., Tissier, A., Balcke, G.U., et al. (2019) The inconspicuous gatekeeper: endophytic *Serendipita vermifera* acts as extended plant protection barrier in the rhizosphere. New Phytol 224: 886–901. 31074884 10.1111/nph.15904

[B32] Sharp, R.G. (2013) A review of the applications of chitin and its derivatives in agriculture to modify plant-microbial interactions and improve crop yields. Agronomy 3: 757–793.

[B33] Sherameti, I., Shahollari, B., Venus, Y., Altschmied, L., Varma, A., and Oelmüller, R. (2005) The endophytic fungus *Piriformospora indica* stimulates the expression of nitrate reductase and the starch-degrading enzyme glucan-water dikinase in tobacco and *Arabidopsis* roots through a homeodomain transcription factor that binds to a conserved motif in their promoters. J Biol Chem 280: 26241–26247. 15710607 10.1074/jbc.M500447200

[B35] Shinya, T., Motoyama, N., Ikeda, A., Wada, M., Kamiya, K., Hayafune, M., et al. (2012) Functional characterization of CEBiP and CERK1 homologs in Arabidopsis and rice reveals the presence of different chitin receptor systems in plants. Plant Cell Physiol 53: 1696–1706. 22891159 10.1093/pcp/pcs113

[B36] Shu, L.-J., Kahlon, P.S., and Ranf, S. (2023) The power of patterns: new insights into pattern-triggered immunity. New Phytol 240: 960–967. 37525301 10.1111/nph.19148

[B37] Takagi, M., Hotamori, K., Naito, K., Matsukawa, S., Egusa, M., Nishizawa, Y., et al. (2022) Chitin-induced systemic disease resistance in rice requires both OsCERK1 and OsCEBiP and is mediated via perturbation of cell-wall biogenesis in leaves. Front Plant Sci 13: 1064628. 36518504 10.3389/fpls.2022.1064628 PMC9742455

[B38] Tominaga, T., Miura, C., Sumigawa, Y., Hirose, Y., Yamaguchi, K., Shigenobu, S., et al. (2021) Conservation and diversity in gibberellin-mediated transcriptional responses among host plants forming distinct arbuscular mycorrhizal morphotypes. Front Plant Sci 12: 795695. 34975984 10.3389/fpls.2021.795695 PMC8718060

[B39] Vishwanathan, K., Zienkiewicz, K., Liu, Y., Janz, D., Feussner, I., Polle, A., and Haney, C.H. (2020) Ectomycorrhizal fungi induce systemic resistance against insects on a nonmycorrhizal plant in a CERK1-dependent manner. New Phytol 228: 728–740. 32473606 10.1111/nph.16715

[B40] Vlot, A.C., Sales, J.H., Lenk, M., Bauer, K., Brambilla, A., Sommer, A., et al. (2021) Systemic propagation of immunity in plants. New Phytol 229: 1234–1250. 32978988 10.1111/nph.16953

[B42] Warcup, J.H. (1988) Mycorrhizal associations of isolates of *Sebacina vermifera*. New Phytol 110: 227–231.

[B43] Yang, C., Wang, E., and Liu, J. (2022) CERK1, more than a co-receptor in plant–microbe interactions. New Phytol 234: 1606–1613. 35297054 10.1111/nph.18074

[B44] Yao, X., Guo, H., Zhang, K., Zhao, M., Ruan, J., and Chen, J. (2023) Trichoderma and its role in biological control of plant fungal and nematode disease. Front Microbiol 14: 1160551. 37206337 10.3389/fmicb.2023.1160551 PMC10189891

[B45] Yuan, M., Ngou, B.P.M., Ding, P., and Xin, X.-F. (2021) PTI-ETI crosstalk: an integrative view of plant immunity. Curr Opin Plant Biol 62: 102030. 33684883 10.1016/j.pbi.2021.102030

