# Supplementary material for: Root Colonization by Trichoderma atroviride Triggers Induced Systemic Resistance Primarily Independent of the Chitin-mediated Signaling Pathway in Arabidopsis
Source: Microbes Environ. 2024 Dec 27;39(4):ME24038. doi: 10.1264/jsme2.ME24038 (PMC11821762; doi:10.1264/jsme2.ME24038)
Supplement: Supplementary file 1 — Supplementary Material 1 [file 39_24038_s1.pdf]

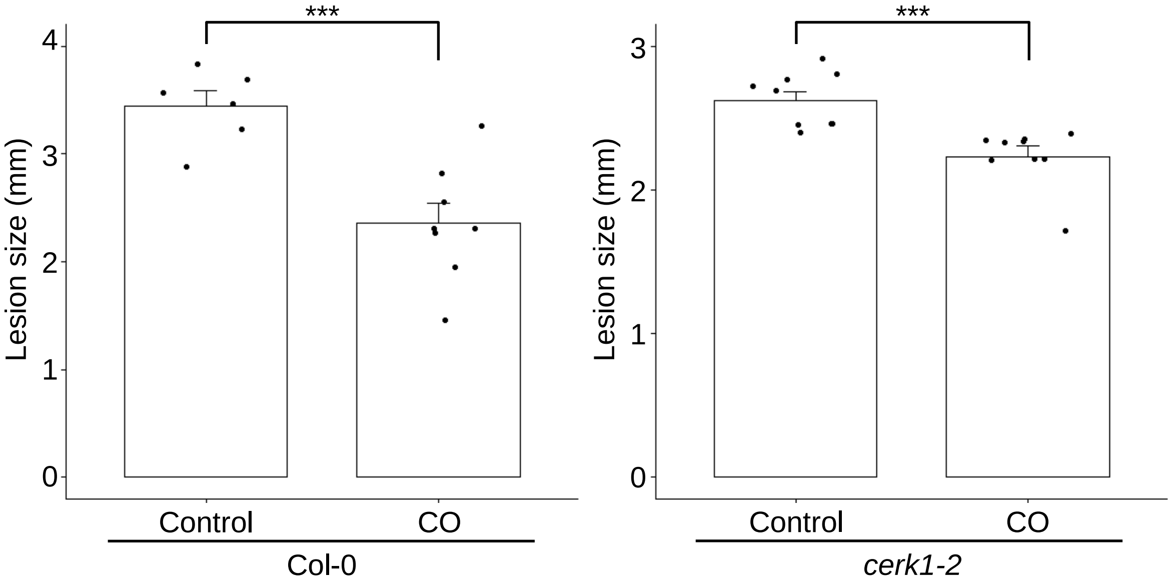

Supplementary Figure 1
Effects of chitin receptor deficiency on induced systemic resistance against a necrotrophic pathogen. Disease resistance against *Alternaria brassicicola* on leaves of wild-type (Col-0) and *cerk1-2* seedlings grown in soils mixed with same volume of 0.1% chitin oligosaccharides (CO) water solution, as conducted in Fig. 2. The bars and error bars indicate means and standard errors, and asterisks indicate statistically significant differences (Student's *t*-test: ***$P < 0.001$; $n \geq 6$).

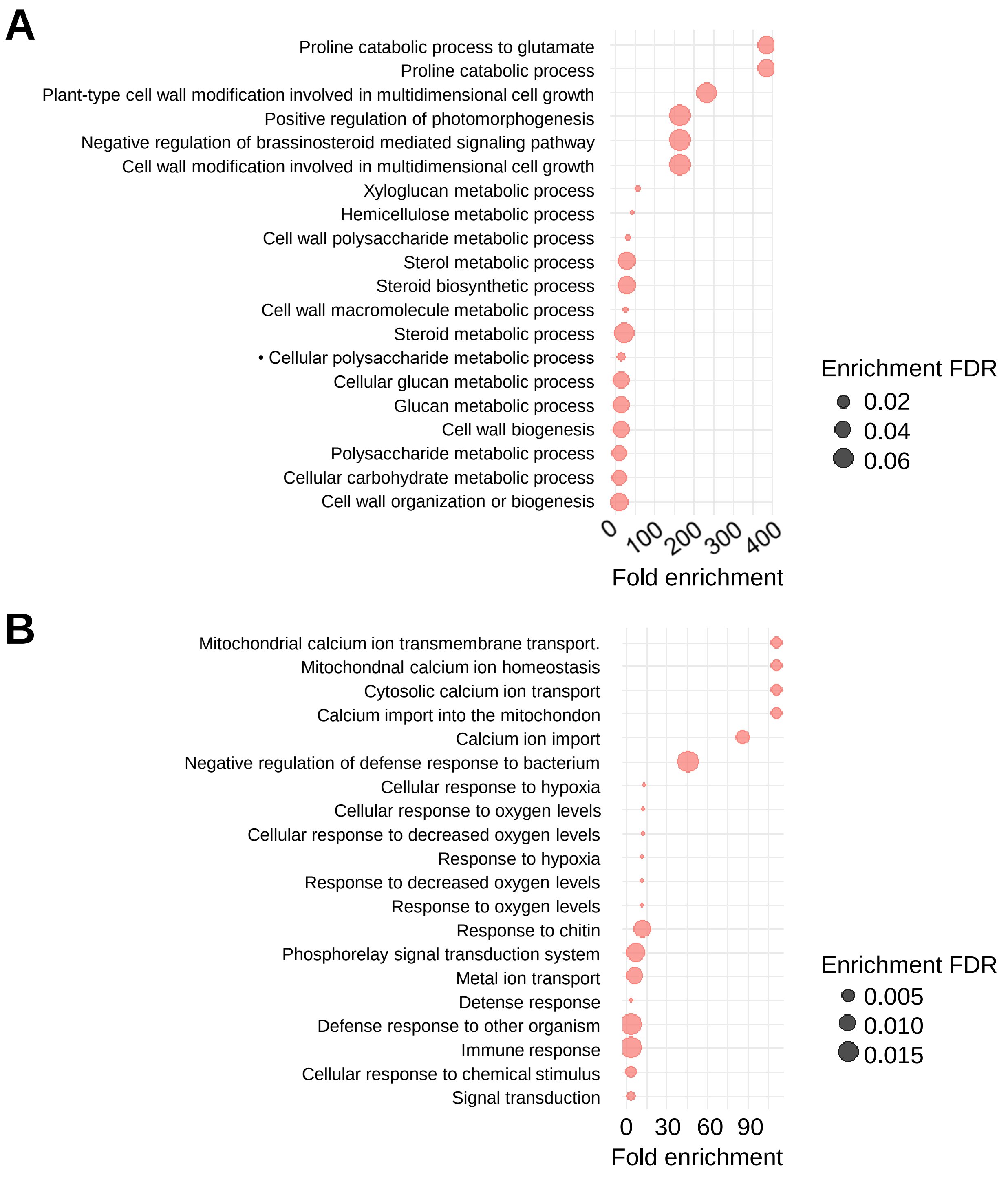

Supplementary Figure 2

GO enrichment analysis of the leaves of Arabidopsis seedlings treated with chitin. GO enrichment analysis of upregulated (A) and downregulated (B) DEGs regulated by chitin treated. The circle size indicates the FDR value. These figures showed the top 20 GO terms with the lowest FDR values in the biological process dataset.
